# Unravelling the dynamics of seed‐stored mRNAs during seed priming

**DOI:** 10.1111/nph.70098

**Published:** 2025-03-28

**Authors:** Patricija Gran, Tessa W. Visscher, Bing Bai, Harm Nijveen, Amir Mahboubi, Lars L. Bakermans, Leo A. J. Willems, Leónie Bentsink

**Affiliations:** ^1^ Wageningen Seed Science Centre, Laboratory of Plant Physiology Wageningen University 6708 PB Wageningen the Netherlands; ^2^ Bioinformatics Group Wageningen University 6708 PB Wageningen the Netherlands; ^3^ Department of Plant Physiology Umeå University SE‐90736 Umeå Sweden

**Keywords:** *Arabidopsis thaliana*, germination, hydropriming, longevity, mRNA dynamics, polysome profiling, RNC‐seq, seeds

## Abstract

- Seed priming is a pre‐sowing treatment that enables more efficient and uniform seed germination; however, it negatively affects seed longevity. In this work, the mRNA dynamics underlying a hydropriming treatment have been investigated.
- Polysome profiling was performed on seeds during different stages of hydropriming. Ribosome nascent chain complex sequencing (RNC‐seq) elucidated transcriptomic and translatomic changes during the priming treatment.
- In contrast to mature dry seeds, hydroprimed seeds contain more mRNA‐ribosome complexes, suggesting that the mRNAs that need to be translated during germination are already associated with ribosomes in the primed seeds, leading to a quicker restart of translation and thus faster germination upon re‐imbibition. As a result of priming, seeds lose part of their stress‐related transcriptome.
- This work highlights genes that might play a role in increasing the rate of germination after priming.

## Abstract 2

See also the Commentary on this article by Sano, **247**: 1971–1973.

## Introduction

**Fig. 1:**
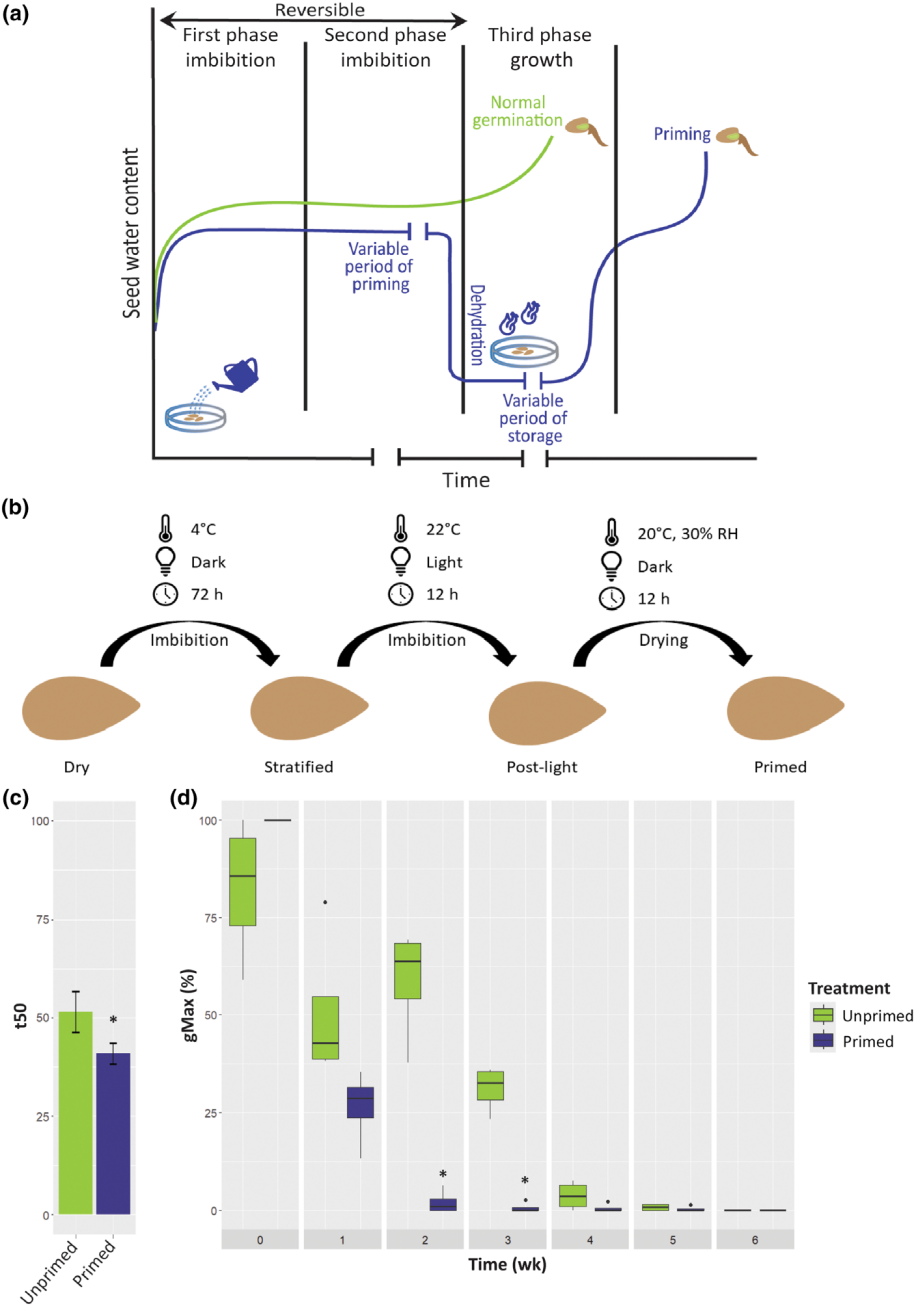
Phenotypic characteristics of primed seeds. (a) Schematic presentation of the priming procedure. Seed water content curves are indicated for normal germination (in green) and for priming (in blue). (b) Schematic representation of the hydropriming protocol used in this research. *Arabidopsis thaliana* seeds underwent controlled imbibition at 4°C in the dark for 72 h (stratification), 22°C in the light for 12 h (post‐light), and subsequent drying at 20°C and 30% relative humidity (RH) for 12 h to complete the priming process. (c) Rate of germination (t50, hours) of unprimed and primed Col‐0 seeds. (d) Germination after artificial ageing (controlled deterioration test) of unprimed and hydroprimed seeds. Mean values and SE are depicted based on four biological replicates. Asterisks denote significance levels determined through *t*‐test: *, *P* ≤ 0.05.

In automated agricultural production, it is important that seed germination occurs quickly and simultaneously. To enhance germination and seedling establishment, even under detrimental environmental conditions, a method called seed priming can be applied. Seed priming is a pre‐sowing seed treatment that aims to enhance seed performance by modulating the physiological and biochemical processes within the seed (Varier *et al*., 2010; Paparella *et al*., 2015). There are different priming methods; however, with respect to the work discussed here, we will focus on hydropriming. Hydropriming is simple, cost‐effective and known to promote the germination rate of Arabidopsis seeds (Sano *et al*., 2017). The germination process, which starts with the hydration of the seed, can be divided into three phases (Bewley *et al*., 2013). The first phase (imbibition/hydration) is characterized by rapid water intake and occurs regardless of the viability or metabolic activity of the seed. This first phase of water absorption is a physical and reversible process; seeds can be re‐dried without losing their germinability. The second (lag) phase is characterized by little water absorption, an increase in enzyme activation, protein synthesis and repair of mitochondria and DNA (Rajjou *et al*., 2012). The third phase includes rapid water intake, radicle protrusion (germination senso stricto), and cell elongation without cell division. Enzymes from the second phase begin to degrade while stored components like fatty acids, proteins, carbohydrates and phosphorus‐containing compounds are consumed by the newly emerging plant. During the priming treatment, the seed is taken through the first two reversible phases of germination and is stopped before the radicle protrudes the endosperm and seed coat (third phase, Fig. 1a). It has been reported that seed priming, in addition to a quicker and more uniform germination, can also improve plant performance in drought and high salinity conditions (Marthandan *et al*., 2020).

Previous studies have shown that seeds accumulate mRNAs during late seed maturation (Rajjou *et al*., 2004; Sano *et al*., 2020). Unlike the majority of RNAs in metabolically active cells, seed‐stored mRNAs are long‐lived, being able to survive for many years (Bai *et al*., 2020; Sano *et al*., 2020). Those long‐lived mRNAs display the ability to survive desiccation and remain translatable in dry quiescent embryos. In order for seeds to germinate, translation of seed‐stored mRNA into proteins has to take place. For translation to happen, seed‐stored mRNAs have to associate with ribosomes. The association of mRNAs with ribosomes can be studied using polysome profiling, a technique that involves the fractionation of RNAs using a sucrose gradient (Mustroph *et al*., 2009). Monosome complexes (mRNAs bound to one ribosome) are present in dry seeds where translation is stalled, while polysome complexes (mRNAs with more than one ribosome attached) accumulate upon seed imbibition (Bai *et al*., 2020; Sano *et al*., 2020). Ribosome nascent chain complex sequencing (RNC‐seq) is a technique that can be used to identify transcripts that are associated with ribosomes and to study translational regulation. The technique relies on the separation of mRNAs bound by various numbers of ribosomes through a sucrose gradient by ultracentrifugation. By comparing the RNC‐seq to total mRNA‐seq performed on the samples, insight into the translation status and dynamics of individual mRNA molecules can be obtained (Zhao *et al*., 2019; Cai *et al*., 2021).

Here, we investigate the dynamics of seed‐stored mRNAs during the priming process to elucidate whether transcriptional or translational changes explain the quicker germination and reduced longevity of primed seeds. mRNA dynamics refers to the changes in the levels, composition and activity of mRNAs throughout the priming process. This includes an understanding of how the abundance of specific mRNAs fluctuates, how they are translated into proteins, and how their levels and activities correlate with the improved germination observed in primed seeds. To determine mRNA dynamics and their association with ribosomes, polysome profiling and RNC‐seq were performed at the consecutive stages during the priming treatment: in dry seeds, stratified seeds, seeds that are exposed to light and the final dried hydroprimed seeds. This work provides insight into mRNA dynamics and molecular processes underlying the hydropriming treatment and highlights the genes that might play a role in faster germination.

## Materials and Methods

### Plant material

For polysome profiling and RNC‐seq, *Arabidopsis thaliana* (L.) Heynh accession Columbia‐0 (Col‐0, N60000) plants were grown on rockwool supplemented with a Hyponex solution (Nitrogen, Phosphorus, Potassium (NPK) = 7 : 6 : 19) in a glasshouse under a long‐day regime (16 h : 8 h, light : dark) at 22°C. Seeds from four biological replicates were harvested in bulk, dried in paper bags at 30% relative humidity (RH) and 20°C for after‐ripening and stored until they reached ≥ 90% germination.

For the investigation of the *RGGB* gene, *A. thaliana* accession Columbia‐0 (Col‐0, N60000), the *rggb* loss‐of‐function mutant (AT4G17520; SALK_040127; N540127), and its complementation line (*pRGGB:RGGB:GFP*) were grown under identical glasshouse conditions (rockwool with Hyponex solution; NPK = 7 : 6 : 19; 22°C, long‐day regime with 16 h : 8 h, light : dark).

The complementation line was generated by amplifying a 3565 base pair genomic fragment, including a 1500 base pair promoter region but lacking the 3′‐UTR, using the forward primer TACATTAGCATCAACGGG and the reverse primer CTTACCCAAAGTAGGACT. The fragment was transferred into the mutant background using the entry vector pDONR207 and destination vector pGWB4 (https://shimane‐u.org/nakagawa/manuals/pGWB_manual_english.pdf) (Supporting Information Table S1). Out of three independent transgenic lines generated, only the one with expression levels closest to Col‐0 was selected for phenotyping (Fig. S1).

To assess expression levels of the Col‐0, the *rggb* loss‐of‐function mutant and the complementation line (pRGGB:RGGB:GFP), total RNA was extracted using a modified hot borate extraction method (Maia *et al*., 2011). Three biological replicates of 5 mg primed seeds were used per sample. cDNA was synthesized from 1000 ng of RNA using the iScript cDNA Synthesis Kit (Bio‐Rad, 170–8890), diluted 10‐fold and 2 μl was used per reaction. Each reaction contained 2.5 μl of SyGreen mix (Sopachem PB20) and 0.5 μl of primer mix (100 μM working stock), with the forward primer TGAAGTCTTCATCAAACTGGGAACAG and the reverse primer CCACCTCTTGGTCTGTAGTAACTCT. Reverse transcription‐quantitative polymerase chain reaction was performed on a Bio‐Rad CFX Opus 384‐well system. Two stable reference genes, At4G12590 and At4G34270, were used for normalization (Dekkers *et al*., 2012). The primer sequences for the reference genes were as follows: At4G12590, forward primer GAGATGAAAATGCCATTGATGAC and reverse primer GCACCCAGACTCTTTGATG; At4G34270, forward primer GTGAAAACTGTTGGAGAGAAGCAA and reverse primer TCAACTGGATACCCTTTCGCA. Relative expression levels were calculated using the ΔΔCt method.

Plants were grown in a randomized complete block design with four replicates per genotype. Seeds from each replicate were harvested in bulk, collected in paper bags, and kept in a drying cabinet at 30% RH and 20°C for after‐ripening until they reached ≥ 90% germination.

### Hydropriming treatment and sample preparation

Seeds of the *A. thaliana* accession Col‐0 were used for all priming experiments. The hydropriming method was modified from Sano *et al*. (2017). In brief, two layers of blue blotter paper (Anchor Paper Co., St Paul, MN, USA) were equilibrated with 40 ml of demineralized water in plastic germination trays (15 × 9 × 21 cm); on top of the blue paper, a white filter paper (Macherey Nagel, Fischer Scientific, Landsmeer, the Netherlands) was placed. Per replicate, 450 mg of seeds was distributed in a thin layer. Germination trays were wrapped in aluminium foil and placed in the fridge at 4°C for 72 h to cold‐stratify the seeds. For polysome profiling and RNC‐seq, stratified seed samples were collected into 50‐ml tubes, snap‐frozen in liquid nitrogen, freeze‐dried, and stored at −80°C. For all other samples, the aluminium foil was removed from the germination trays, after which they were packed in a closed transparent plastic bag. The trays were then placed in a 22°C incubator under continuous light (143 μmol m^−2^ s^−1^) for 12 h. The post‐light samples were also collected for polysome profiling and RNC‐seq after 12 h of incubation in these conditions, processed, and stored as described above. The last step of hydropriming involved drying the seeds. Drying was performed by transferring the white filter paper with seeds to a new tray with two layers of blue filter paper. Trays were then placed in a drying cabinet at 30% RH and 20°C for 12 h. To validate the effect of the drying, three technical replicates of both dry (200 mg) and primed seeds were weighed. The average weights of the dry and primed seeds do not significantly differ from each other (unprimed, dry seeds 200 mg, dried primed seeds 193.57 mg (*P*‐value 0.3, *n* = 3)), confirming that they have been sufficiently dried back. Primed samples were then handled again in the same manner as those above for RNC‐seq and polysome profiling, while samples for germination assays were immediately used or stored at 30% RH and 20°C.

### Seed germination assay

Two layers of blue blotter paper (Anchor Paper Co., St Paul, MN, USA) were equilibrated with 40 ml of demineralized water in plastic trays (15 × 9 × 21 cm), and four biological replicates per genotype of *c*. 50–150 seeds were carefully sown on wetted papers using a mask to ensure accurate spacing. Piled‐up trays were wrapped in a closed transparent plastic bag. The experiment was carried out in a 22°C incubator under continuous light (143 μmol m^−2^ s^−1^). Pictures were taken multiple times a day for a period of 7 d using a Nikon D80 camera (Nikon, Tokyo, Japan) fixed to a repro stand with a 60‐mm macro‐objective. The camera was connected to a computer using the Nikon Camera Control Pro software, v.2.0. Germination was scored using the germinator package (Joosen *et al*., 2010). To determine the days of seed dry storage required for 50% germination (DSDS50) values (Alonso‐Blanco *et al*., 2003; He *et al*., 2014; Soppe & Bentsink, 2020), germination assays were carried out from 3 d until 5 wk after harvest, when the seeds were fully after‐ripened (> 90% germination). The germination experiments were performed as described above. The DSDS50 levels were calculated using the statistical program R v.2.14 (R Development Core Team, 2009; www.rproject.org) (He *et al*., 2014). The script is provided in Notes S1.

To test germination performance in suboptimal conditions, two layers of blue blotter paper (Anchor Paper Co., St Paul, MN, USA) were equilibrated with 40 ml of 200 mM D‐mannitol, 50 mM NaCl and water in plastic trays (15 × 9 × 21 cm). The germination assay was performed as described above.

### Seed longevity assay

Seed longevity was assessed via a controlled deterioration test (CDT) performed as described by Righetti *et al*. (2015). The seeds were exposed to 38°C and RH of 75%. For each line, *c*. 50–100 seeds (per replicate) were put in 2‐ml Eppendorf tubes and placed in the sealed plastic box. The RH level of 75% was maintained within the box by placing two trays of saturated salt (NaCl) at the bottom of the box. The box was placed in a dark climate chamber for 6 wk. The experiment was performed with four replicates per line. The germination potential of aged seeds was assessed through germination assays as described above. The p50 values, representing the time at which 50% of the seed population has lost viability, were determined using a DSDS50 calculation method. The analysis was performed in R (v.2.14; R Development Core Team, 2009) using the DSDS50 script. A germination curve was fitted to viability decline data, and the time at which seed viability reached 50% was extracted. The full R script is provided in Notes S1.

### Polysome profiling

Polysome extraction was modified from Mustroph *et al*. (2009). In detail, per replicate (total three replicates were analysed) 300 mg of freeze‐dried seed sample was ground using a mortar and pestle and dissolved in 4 ml of polysome extraction buffer (200 mM Tris–HCl (pH 8.0), 200 mM KCl, 35 mM MgCl₂ × 6H₂O, 25 mM egtazic acid (ethylene glycol‐bis(β‐aminoethyl ether)‐N,N,N′,N′‐tetraacetic acid (EGTA)), 1 mM dithiothreitol (DTT), 1 mM phenylmethanesulfonylfluoride, 100 μg ml^−1^ cycloheximide, 1% (v/v) polyoxyethylene 10 tridecyl ether) and kept on ice for 10 min. The seed debris was removed by centrifuging at 16 000 ***g*** for 15 min at 4°C in a JA‐25.50 rotor and the Avanti J‐20 XP centrifuge (Beckman Coulter, Fullerton, CA, USA). The clear supernatant was then moved to a new tube and diluted with an additional 4 ml of polysome extraction buffer. Samples were then gently placed on top of an 8‐ml sucrose cushion (100 mM Tris–HCl (pH 8.0), 40 mM KCl, 20 mM MgCl₂ × 6H₂O, 5 mM EGTA, 1 mM DTT, 100 μg ml^−1^ cycloheximide in 60% sucrose) in a 26‐ml polycarbonate tube (Beckman Coulter). Samples were balanced and centrifuged at 90 000 ***g*** for 18 h at 4°C in a 70Ti rotor and L8‐M ultracentrifuge (Beckman Coulter). After centrifugation, the supernatant was removed and the polysome pellets were washed with MilliQ water and resuspended in 400 μl of resuspension buffer (100 mM Tris–HCl (pH 8.0), 40 mM KCl, 20 mM MgCl₂ × 6H₂O, 100 μg ml^−1^ cycloheximide). The resuspended samples were kept on ice for 30 min followed by centrifugation at 16 000 ***g*** at 4°C to remove any debris. The RNA content was measured for each sample using the Qubit BR RNA assay kit (Thermo Fisher Scientific, Waltham, MA, USA). Equal quantities of ribosomes, determined by their optical density at 254 nm, were then loaded on 15–60% sucrose gradients and centrifuged at 190 000 ***g*** in a SW55 Ti rotor and L8‐M ultracentrifuge (Beckman Coulter). The gradient samples were fractionated using an Teledyne ISCO absorbance detector (model # UA‐5; ISCO, Lincoln, NE, USA) to obtain ribosome‐containing fractions. The presented profiles were generated by subtracting the baseline obtained by measuring a blank gradient. The total area under the monosome (mRNA associated to one ribosome), disome (mRNA associated to two ribosomes), and polysome (mRNA associated to more than two ribosomes) peaks was calculated for each priming stage across three biological replicates in R (Notes S1). Peak quantification was performed to assess the abundance of ribosome‐mRNA complexes at different stages of priming. Normalization was conducted by first calculating the total area under each individual peak (monosome, disome, and polysome) and then determining their relative contributions by dividing the individual peak areas by the total peak area.

### Isolation of total mRNA and ribosome associated mRNA

Total RNA was isolated as described by Bai *et al*. (2017) with minor modifications. In detail, per replicate (total three replicates were analysed) 450 mg dry weight of freeze‐dried seed material was subjected to extraction with 10 ml of polysome extraction buffer (PEB: 400 mM Tris pH 9.0, 0.25 M sucrose, 200 mM KCl, 35 mM MgCl₂ × 6H₂O, 5 mM EGTA, 1 mM phenylmethane sulfonyl fluoride, 5 mM DTT, 50 μg ml^−1^ cycloheximide and 50 μg ml^−1^ chloramphenicol). The extracts, 10 ml in volume, were gently loaded on top of an 8 ml sucrose cushion (1.75 M sucrose in PEB) and centrifuged (18 h, 90 000 ***g***) using a Beckman Ti50.2 rotor at 4°C (Beckman Coulter, Brea, CA, USA). After centrifugation, at the bottom of the tubes, the pellet was formed. The pellet was then resuspended in wash buffer (200 mM Tris pH 9.0, 200 mM KCl, 0.025 M EGTA, 35 mM MgCl₂ × 6H₂O, 5 mM DTT, 50 μg ml^−1^ cycloheximide, 50 μg ml^−1^ chloramphenicol) to obtain ribosome‐associated RNA. These samples were used for isolating the RNC RNA. The aliquots of total and ribosome‐associated RNA were further prepared for RNA sequencing using the clean‐up RNeasy Mini Kit (Qiagen).

Sequencing: all RNA samples were sequenced with the Illumina NovaSeq 6000 platform (Illumina, Inc., San Diego, CA, USA), following the PE150 strategy for unstranded sequencing. For this, the RNA samples were first fragmented into pieces of *c*. 250 bp. RNA fragments were reverse‐transcribed to cDNA with random hexamer primers, after which the cDNAs were dA‐tailed. The dA‐tailed DNA fragments were then ligated to sequencing adaptors. The final DNA library was obtained with PCR amplification. Sequencing data generated in this study have been deposited in the public data archive, the Sequence Read Archive (SRA) under BioProject accession number PRJNA1153751.

### Quality control, alignment and read counting

All data analysis steps were performed on in‐house Linux servers. Analyses that required an R package were run in Rstudio Server (v.1.3.1093; R v.4.0.4 (2021‐02‐15)). For the read pairs, they were aligned to the Arabidopsis The Arabidopsis Information Resource, version 10 (TAIR10) genome using the R package Rsubread with default settings (v.2.4.2; Liao *et al*., 2019). Read counts were assigned to exons with the R package featureCounts using the Araport 11 (Cheng *et al*., 2017) Arabidopsis genome annotation (featureType = ‘exon’, countMultiMappingReads = FALSE, PairedEnd = TRUE). The Arabidopsis TAIR10 reference genome and Araport11 annotation were downloaded from the Arabidopsis Information Resource (https://www.arabidopsis.org/).

### Principal component analysis (PCA)

To perform the PCA analysis, the R package DESeq2 (v.1.30.0; Love *et al*., 2014) was used. First, a DESeqDataset object was created from the filtered read counts. The counts were rlog()transformed and used as input for the DESeq2:::plotPCA function (using default settings). The R package ggplot2 (v.3.3.3; Wickham, 2009) was used for the visualization of the PCA plot.

### Identification of differentially expressed and translationally regulated genes

To identify differentially expressed and translationally regulated genes, R package DESeq2 (v. 1.30.0; Love *et al*., 2014) was run with the likelihood ratio test after which the log_2_ fold changes (LFC) were shrunk by running lfcShrink with ashr as shrinkage estimator (Stephens, 2016). For the identification of both the differentially expressed and translationally regulated genes, the consecutive priming stages (dry to stratified, stratified to post‐light, and post‐light to primed) and the first and last priming stage (dry to primed) were compared. For each comparison, the earliest stage was used as reference. Genes were identified as differentially expressed if their transcript levels (based on RNA‐seq data) between two priming stages were significantly different (*P*adj < 0.05) (Benjamini & Hochberg, 1995). Genes were classified as translationally regulated if their association to ribosomes (based on RNC‐seq data) changed independently and significantly (*P* < 0.05) when compared to the transcript levels (RNA‐Seq) or vice versa. For this, an interaction term between the sequencing type (RNC‐seq or RNA‐seq) and priming stage (dry, stratified, post‐light or primed) was included in the linear model design of the DESeq2 object (~Stage + SeqType + Stage:SeqType). This approach to identify translationally regulated genes has previously been described (Chothani *et al*., 2019).

### Gene ontology enrichment

For the gene ontology (GO) enrichment analyses, the R package clusterProfiler was used (v.3.18.1; Yu *et al*., 2012). The R package AnnotationHub (v.2.22.1; Morgan & Shepherd, 2021) was first used to retrieve the org.At.tair.db.sqlite database (AH84114) to map the Arabidopsis Genome Initiative (AGI) codes to the GO terms. GO terms with an adjusted *P*‐value < 0.05 were considered as significantly enriched. The top 5 enriched GO terms were visualized for each gene set with ggplot2.

### STRING‐DB

A functional association network for the list of putative regulators was built using Search Tool for the Retrieval of Interacting Genes/Proteins Database (STRING‐DB) (http://string‐db.org; Szklarczyk *et al*., 2019). For this, a full STRING network with a medium confidence of 0.400 and a medium false discovery rate stringency of 5% was built. Disconnected nodes were removed from the analysis.

## Results

### Phenotypic characteristics of primed seeds

Hydropriming involves the controlled hydration of seeds to initiate the early stages of germination, without allowing the radicle protrusion (Fig. 1a). The hydropriming protocol used in this study involved controlled imbibition of seeds at 4°C in the dark for 72 h (stratification). This was followed by 12 h at 22°C under light (post‐light treatment). Finally, the seeds were dried at 20°C and 30% relative humidity for 12 h to complete the priming process (Fig. 1b). Hydropriming is known to speed up germination and increase uniformity (Khalid *et al*., 2019). To confirm these priming effects, hydroprimed seeds were tested for their germination performance. Primed seeds reached 50% of germination *c*. 20 h earlier and germinated to a higher percentage in mannitol than their unprimed counterparts. The maximum germination (gMAX), area under the curve (AUC), uniformity in control conditions and the maximum germination in salt were not significantly improved, although the variation in these traits was less in the primed seeds (Figs 1c, S2). To confirm the negative effect of hydropriming on seed longevity, seeds were artificially aged using a mild CDT. The germination performance of primed seeds significantly decreased compared with that of unprimed seeds after a week of exposure to 75% RH and 38°C (Fig. 1d).

### Primed seeds contain more mRNA‐ribosome complexes

**Fig. 2:**
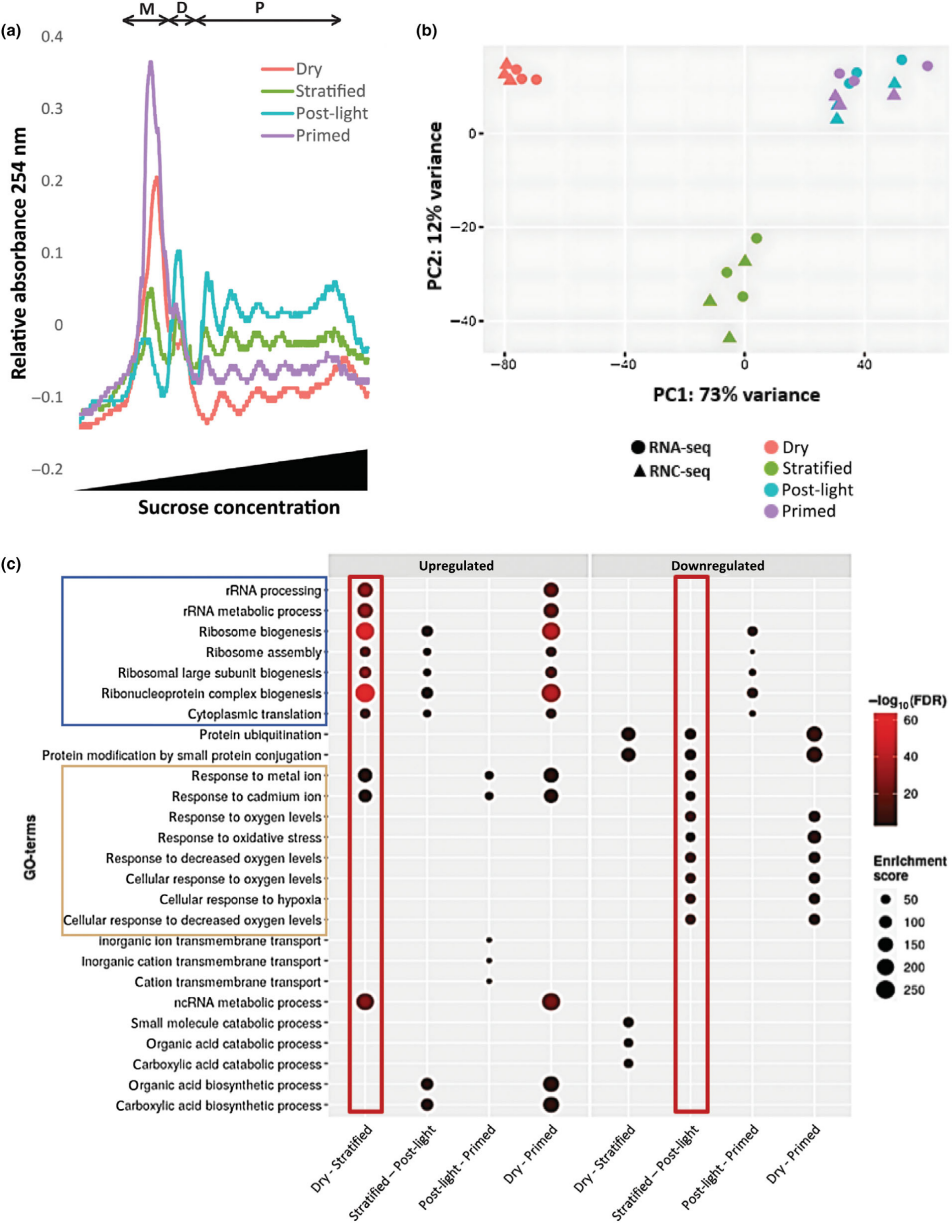
Dry and primed seeds can be discriminated by their transcriptome profiles. (a) Polysome profiles of *Arabidopsis thaliana* seeds during different stages of hydropriming. Representative absorbance profiles of sucrose density gradient fractionated ribosomes for dry (salmon), stratified (green), post‐light (turquoise), and primed (purple) seed samples. Peaks in the monosome area (M), disome (D), and the polysome area (P) are presented. Experiment was performed with three technical replicates per stage. (b) Principal component (PC) analysis on RNA‐seq (circles) and ribosome nascent chain complex sequencing (RNC‐seq) (triangles) samples at different priming stages, dry (salmon), stratified (green), post‐light (turquoise), and primed (purple). (c) Gene ontology (GO) terms that are up‐ and downregulated when comparing a priming stage to the next stage, dry unprimed seeds vs 12 h stratified seeds (stratified), stratified vs 12 h light treatment (post‐light), post‐light vs final dried primed seeds (primed), and dry unprimed seeds vs dried primed seeds. The size of the dots indicates the number of genes (which refers to the number of genes that is associated with this GO term). The colour of the dots indicates the significance of the up‐ or downregulation. Red rectangles indicate the transition from dry to stratified stage with the most upregulated GO terms related to translational efficiency and downregulated GO terms related to several stress responses in the shift from the stratified to the post‐light stage. The blue rectangle indicates GO terms related to translation and the beige rectangle indicates GO terms related to stress responses.

To determine mRNA dynamics and their association with ribosomes, polysome profiling was performed at four consecutive priming stages: dry seeds (starting material before priming, dry), seeds after 12 h of stratification (stratified), seeds after 12 h of stratification and 12 h light treatment (post‐light) and the final dried primed seeds (primed) (three technical replicates per stage) (Fig. 2a). The changes in ribosome association were quantified by calculating the total area as well as the relative average area under the monosome, disome and polysome peaks across three biological replicates (Fig. S3). Once water becomes available during cold stratification, but other key germination factors like light and temperature are not optimal, the recruitment of polysomes at the cost of monosomes was observed. Upon the availability of light and increased temperature (22°C), there is an increase in total mRNA‐ribosome complexes, indicating that new ribosomes are synthesized. When comparing primed seeds to dry seeds, it becomes clear that the total amount of ribosome‐mRNA complexes significantly increased; however, the quantification showed that there are no significant changes in the monosome, disome and polysome ratios between dry and primed seeds (Fig. S3).

### Hydropriming leads to the loss of the stress‐related transcriptome

Total mRNA (RNA‐seq) and ribosome associated mRNA (RNC‐seq) levels were measured to investigate the mRNA dynamics at the four consecutive priming stages, dry seeds (starting material before priming, dry), seeds after 12 h of stratification (stratified), seeds after 12 h of stratification and 12 h light treatment (post‐light), and the final dried primed seeds (primed). The main distinction between RNA‐seq and RNC‐seq lies in the type of RNA molecules being analysed. While RNA‐seq examines the total RNA population, including both translated and untranslated RNAs, RNC‐seq specifically targets the RNA transcripts that are actively engaged with ribosomes providing valuable information about the translational status of specific RNA transcripts. After filtering out the low expressed genes, 14 642 genes were identified over all priming stages and 14 320 of these referred to protein coding transcripts. To visualize differences/similarities between the different stages of priming, a principal component analysis (PCA) on the log‐transformed data was performed (Fig. 2b). The PCA of the first two components showed that samples from the dry and stratified stage formed distinct groups. However, no clear difference was visible between the total mRNA and ribosome‐associated mRNA samples. In addition, samples from the post‐light and primed stage clustered together, suggesting that drying seeds back causes fewer changes. To identify translationally active genes that differ in abundance between the consecutive priming stages, Deseq2 analyses were performed on the RNC‐mRNA. In addition, the first stage (dry mature seeds) was compared with the last stage (primed seeds) of the priming treatment, since differences between these stages might explain the differences in the phenotypic behaviour of primed and unprimed seeds (Fig. 1). For each comparison, changes in the later stage were identified relative to the earlier stage. In total, between all the stages of the hydropriming treatment, 4454 genes were exclusively RNC upregulated (meaning that these were not downregulated at any of the stages) and 2789 genes were exclusively RNC downregulated (Table 1). The transition from post‐light to primed seeds showed the lowest number of upregulated genes (714), while the transition from stratified to post‐light showed the lowest number of 943 downregulated genes. During the shift from dry to stratified stage, 3553 genes were upregulated and 2272 genes were downregulated. The stratified to post‐light stage showed 1423 upregulated genes. In transition from post‐light to primed, 1216 genes were downregulated. To analyse the overall function of genes being up‐ or downregulated between different priming stages, GO analyses were performed. The most noticeable finding was that biological process GO terms related to translational efficiency, including terms related to rRNA and ribosomes, were significantly upregulated in the RNC‐mRNA during all priming steps except for the post‐light to the primed stage, where these processes were significantly downregulated (Fig. 2c). Another striking finding was that GO terms related to several stress responses, including metal and oxygen stress, were significantly RNC‐downregulated in the shift from the stratified to the post‐light stage. This was also reflected in the primed stage, where genes related to GO stress terms were significantly lower than in the dry stage. These results suggest that seeds lose part of their ability to respond to stresses during stratification and that this is intensified by the exposure to light.

**Table 1:** Overview of *Arabidopsis thaliana* transcripts that differ in abundance between the consecutive priming stages.

	Stratified vs dry	Post‐light vs stratified	Primed vs post‐light	Primed vs dry
Ribosome‐associated mRNA				
Upregulated	3553	1423	714	4545
Downregulated	2272	943	1216	2395
Total mRNA				
Upregulated	3667	1838	567	4451
Downregulated	2700	1362	740	3107
Translational regulation				
Upregulated	180	0	15	286
Downregulated	45	1	73	59

Number of ribosome‐associated, total, and translationally regulated mRNAs between hydropriming stages.

### Translational regulation during seed priming

**Fig. 3:**
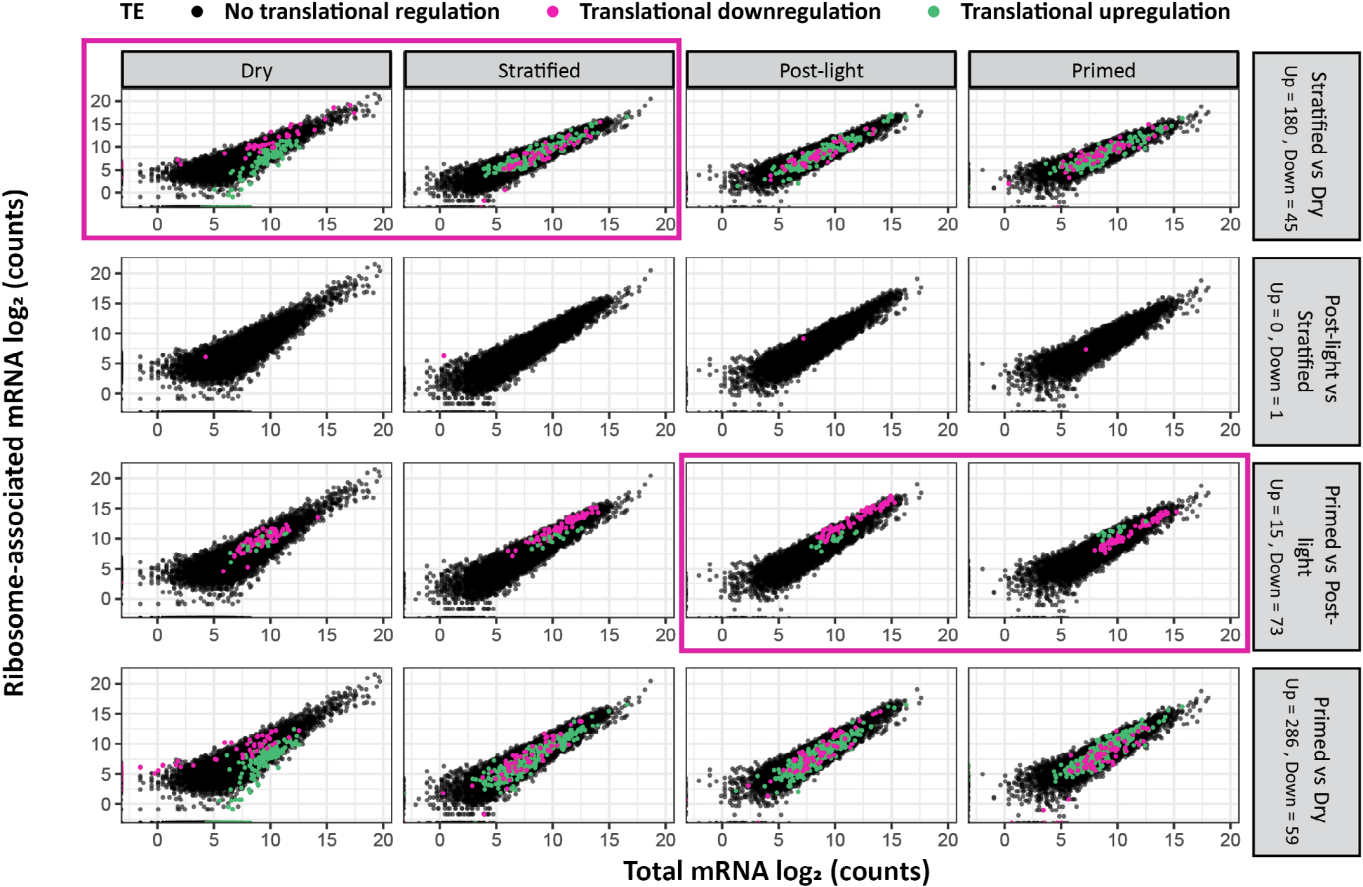
Two distinct stages with changes in translational regulation during seed priming. The counts of the *Arabidopsis thaliana* total mRNA abundance (*x*‐axis) and ribosomal‐associated mRNA counts (*y*‐axis) in the four investigated stages (columns: dry, stratified, post‐light, and primed) are plotted. In each row, the same set of genes from a specific comparison is displayed across the different stages to highlight stage‐specific changes in translational regulation. Grey boxes on the right indicate the number of transcripts that are translationally up‐ or downregulated when comparing the indicated consecutive stages. Genes that are translationally upregulated are visualized in green, while genes that are translationally downregulated are visualized in magenta. Black dots show genes that are not translationally regulated.

It is known that during seed germination there is a lot of translational regulation (Galland *et al*., 2014; Bai *et al*., 2017). To understand what is happening during the priming treatment at the level of translationally regulated genes, the consecutive priming stages (dry to stratified, stratified to post‐light, and post‐light to primed) and dry to primed were compared. Genes were classified as translationally regulated if their ribosomal association (based on RNC‐seq data) changed independently of the transcript levels (based on RNA‐seq data) or vice versa. If the RNC‐mRNA counts increased compared with the total mRNA counts, genes were identified as translationally upregulated. If they showed the opposite pattern, genes were identified as translationally downregulated. The comparison was made between seed stages, with the ratios compared for each stage (Fig. 3). Two stages of translational regulation were identified. A severe translational regulation was revealed when comparing dry to stratified shift; 180 genes were translationally upregulated and 45 genes were translationally downregulated. Remarkably, many of these genes are expressed as free transcripts in the dry seed; 122 out of 231 (green dots at the *x*‐axis; Fig. 3 upper left panel). Among the translationally upregulated genes in the stratified seeds are 18 genes that depend on ATP activity, like RNA helicases. Furthermore, there are 21 genes that are described to be RNA binding. The translationally downregulated genes in the stratified stage are mostly related to seed maturation, like the Late Embryogenesis Abundant (LEA) genes (AT1G52690, AT2G21490, AT2G23120, AT3G02480, and AT4G21020) and *DOGL4*. The second stage at which translational regulation was identified was from the post‐light to primed shift, where 15 genes were translationally upregulated and 73 genes downregulated. The translationally upregulated genes at this stage do not represent a specific class of genes, whereas the downregulated genes are almost exclusively related to translation. This suggests that translation is downregulated in primed seeds, which is confirmed also by the polysome profiles. From the stratified to the post‐light stage, there was basically no translational regulation, except for one gene that was translationally (down)regulated (AT1G32310).

What should be noted as well is that 286 genes were translationally upregulated and 59 genes were translationally downregulated when comparing the primed to dry seeds. This suggests that 286 genes became associated with ribosomes during the priming process that were not translationally downregulated during seed drying. Likely, these genes play a role in the increased germination of primed seeds. Of these transcripts, 106 are already translationally upregulated from stratification onwards. These include not only the earlier identified genes for stratification, such as upregulated ATP activity‐related genes like RNA helicases, but also two genes from the type 2C protein phosphatases: one from the clade D family and the other from clade H (Dataset S1) (Akiyama *et al*., 2022). Moreover, this gene set includes transcripts of several RNA‐binding proteins (RBPs) (Table 2). These results suggest that translational regulation plays a crucial role during seed germination. We hypothesize that by interacting with specific mRNAs and modulating their stability, translation and processing, RBPs play an important role in orchestrating the complex gene expression networks that drive the germination process.

**Table 2:** Overview of translationally upregulated RNA‐binding proteins in primed seeds.

Translationally upregulated RNA‐binding proteins			
Gene	Name	Role	Reference
AT5G61960	MEI2‐LIKE PROTEIN 1 (ML1)	Involvement in regulating gene expression and maintaining RNA stability under desiccation conditions	Masaki *et al*. (2008)
AT4G11420	EUKARYOTIC TRANSLATION INITIATION FACTOR 3A (EIF3A)	Coordinating the assembly of the translation initiation complex and promoting the recruitment of the ribosome to the mRNA transcript	Raabe *et al*. (2019)
AT5G58040	HOMOLOG OF YEAST FIP1	Fip1 homolog in Arabidopsis plays a role in RNA binding and is involved in the polyadenylation machinery	Li *et al*. (2023)
AT5G20320	Dicer‐like 4 (DCL4)	Essential for generating siRNAs that are involved in plant defence and immune responses	Li *et al*. (2012)
AT4G32720	LA PROTEIN 1 (La1)	Plays a role in RNA binding and is involved in the polyadenylation machinery	Li *et al*. (2019)
AT5G47210	Hyaluronan/mRNA binding protein 1	Role in inhibiting germination	Sajeev *et al*. (2022)
AT5G07350	TUDOR‐SN PROTEIN 1 (TUDOR1)	Modulating the dynamics of stress granules, which are cytoplasmic structures formed in response to cellular stress	Gao *et al*. (2015)
AT1G13190	RNA‐binding (RRM/RBD/RNP motifs) family protein	Seed‐specific transcriptional regulation, synthesis of seed storage proteins critical for seed maturation and germination	Lara *et al*. (2003); Le *et al*. (2010)
AT2G22100	mTERF18	Involved in regulating heat tolerance and oxidative stress responses	Kim *et al*. (2021)
AT2G44710	RNA‐binding (RRM/RBD/RNP motifs) family protein	May contribute to the mechanisms underlying thermotolerance during seed imbibition and germination	Ribeiro *et al*. (2018)
AT3G14450	CTC‐INTERACTING DOMAIN 9 (CID9)	Contains PAM PABC binding domain involved in post‐transcriptional regulation, mRNA processing and translation termination	Funakoshi *et al*. (2007)

This table lists RNA‐binding proteins (RBPs) associated with genes exhibiting translational upregulation as seeds transition from a dry to a primed state. Inclusion criteria required a statistically significant increase in ribosomal association, determined by an adjusted *P*‐value < 0.05 and a LFC > 0, indicating enhanced association with ribosomes in primed seeds across all levels of LFC. Indicated are the gene ATG numbers, names, their proposed roles as described in the literature and the respective reference.

### The ARGININE‐GLYCINE–GLYCINE RNA BINDING PROTEIN B affects the rate of seed germination

**Fig. 4:**
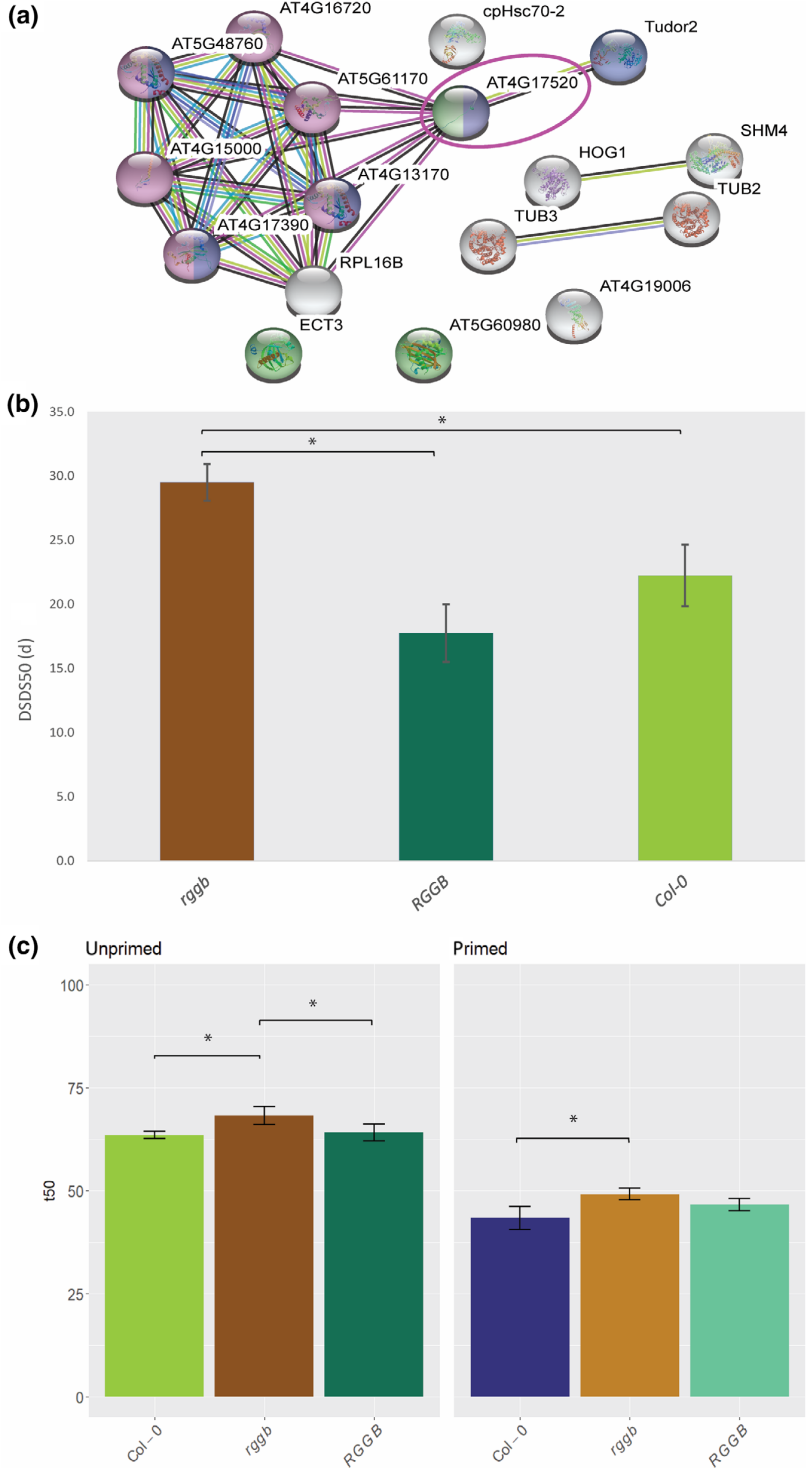
*Arabidopsis thaliana* ARGININE‐GLYCINE–GLYCINE RNA BINDING PROTEIN B (RGGB) affects germination rate and dormancy levels. (a) Protein–protein interactions obtained from STRING‐DB for genes exhibiting a high increased ribosome association (LFC > 5) in primed seeds. Green colour indicates that the protein is part of the ribosome. Blue colour indicates RNA‐binding capacity; purple colour indicates that the protein is involved in translation. The purple circle indicates RGGB (AT4G17520) and its connection to other proteins. (b) Dormancy levels of the *rggb* T‐DNA insertion mutant, its corresponding complementation line RGGB, and Col‐0 were measured as days of seed dry storage required for 50% germination (DSDS50; Alonso‐Blanco *et al*., 2003; Soppe & Bentsink, 2020) He *et al*. (2014). (c) Germination phenotype of RGGB. The rate of germination (t50 in hours) for unprimed and primed seeds of Col‐0, the *rggb* T‐DNA insertion mutant, and its corresponding complementation line. Mean values and SE are depicted based on four biological replicates. Asterisks denote significance levels determined through one‐way ANOVA: *, *P* ≤ 0.05.

We hypothesize that the translation of mRNAs that show an increased association to ribosomes in primed seeds is important for the faster germination of primed seeds compared to that of unprimed dry seeds. Moreover, we were interested in investigating whether the suggested coordination of translation of multiple ribosome‐associated mRNAs could point towards associated functions. Therefore, we identified a set of 30 genes exhibiting a high increased ribosome association (LFC > 5) in primed seeds (Dataset S1) and analysed the potential connections between these proteins by building a functional association network using STRING‐DB (http://stringdb.org; Szklarczyk *et al*., 2019; Fig. 4a). We did not identify protein–protein interactions among the 30 genes that showed increased ribosome association. However, our analysis highlighted the ARGININE‐GLYCINE–GLYCINE RNA BINDING PROTEIN B (RGGB; AT4G17520, also referred to in literature as a member of the Hyaluronan/mRNA binding family) as one of the hubs in a network of genes that are related to mRNA translation (Fig. 4a). A member of the same family, *RGGC*, was identified as RNA binding during seed germination and mutating this gene leads to a reduction in seed dormancy (Sajeev *et al*., 2022). To investigate whether *RGGB* has a similar effect on seed germination, seed dormancy levels, and the germination behaviour of unprimed and primed seeds of the *rggb* knock‐out mutant and its complementation line were analysed (Figs 4, S4). The *rggb* knock‐out mutant is more dormant than its wild‐type Col‐0, and this phenotype is complemented in the line containing the *RGGB* transgene (Fig. 4b). Moreover, both unprimed and primed *rggb* seeds demonstrated significantly slower germination than Col‐0 and the corresponding unprimed *RGGB* complementation line, suggesting that indeed this protein plays a role in determining the germination rate (Fig. 4c). This finding is consistent with a previously suggested aberrant germination phenotype of a T‐DNA line containing an insertion in *RGGB* (or HYALURONAN/mRNA BINDING FAMILY PROTEIN (HLN); At4G17520) (Warmerdam *et al*., 2019). Priming significantly enhanced seed performance across all lines. Primed seeds of Col‐0, the *rggb* mutant and the *RGGB* transgenic line showed increased maximum germination (gMax) and AUC, with statistically significant improvements compared to unprimed seeds for Col‐0 and *RGGB*. Additionally, seed longevity (p50) after controlled deterioration declined with priming for all lines (Fig. S4). There is no significant difference in seed longevity between Col‐0, the *rggb* mutant and the *RGGB* transgenic line.

## Discussion

This study delved into the complexity of mRNA dynamics during the hydropriming treatment of *A. thaliana* seeds. Hydropriming is a widely employed pre‐sowing technique in agriculture, known for its ability to enhance seed germination efficiency, thereby ensuring synchronized germination even under adverse environmental conditions. However, a drawback of this treatment is its negative impact on the longevity of the seeds (Fabrissin *et al*., 2021). By subjecting seeds to a hydropriming treatment, we have observed faster germination and reduced longevity, as reported for Arabidopsis previously by Sano *et al*. (2017).

To unravel the underlying mechanisms of the observed effects of hydropriming, this research utilized advanced techniques such as polysome profiling and RNC‐seq. These methods enabled the comprehensive investigation of changes in the seed transcriptome and translatome (ribosome associated transcripts) throughout different stages of the hydropriming process. In addition to shedding light on the mRNA dynamics and molecular processes occurring during the hydropriming treatment, it also emphasized specific genes that may play a pivotal role in expediting germination. The insights gained from this research have practical implications for optimizing seed germination in agriculture, particularly in automated production systems. From our RNC‐seq data, we can infer that priming has a dual effect on seed physiology: it enhances the translational activity while concurrently diminishing stress responsiveness. During the lag phase of germination, there is an upregulation in the expression of genes that encode ribosomal proteins, leading to an increase in ribosomal activity. This facilitates the *de novo* synthesis of proteins that are required for seed germination (Fu *et al*., 2005; Galland *et al*., 2014). Our study reveals a similar process during seed priming, with differential expression of several genes associated with translational efficiency. Notably, GO terms related to rRNA and ribosomes are significantly upregulated during priming steps, except for the post‐light to primed step, where they are downregulated (Fig. 2c). This suggests that priming accelerates germination primarily by enhancing translational activity. When the germination process is halted in the primed stage, some of these changes are reverted. Our findings indicate that priming increases the total number of ribosome–mRNA complexes while maintaining the monosome, disome, and polysome ratios. The presence of more ribosomes in primed seeds than in dry seeds suggests that priming facilitates translation initiation, which may contribute to the accelerated germination observed in primed seeds. Visual inspection of the profiles (Figs 2a, S3) reveals a higher polysome area in two of the three replicates, whereas the third replicate shows a slightly lower polysome area but a higher disome area. This suggests that ribosome recruitment is a transient process, which stresses the importance of analysing multiple replicates and performing quantitative analyses. Overall, our results suggest that seed priming influences ribosome homeostasis, increasing the total ribosome pool without altering the relative distribution of ribosome‐mRNA complexes. The fact that so many transcriptional changes during priming are related to translational efficiency also underlines the importance of translation during germination (Bai *et al*., 2020; Sano *et al*., 2020). A noteworthy observation is that genes downregulated during the transition from the stratified to post‐light stages are enriched in GO terms related to various stress responses, including metal and oxygen stress. This implies that seeds lose some of their ability to respond to stresses during seed priming, exacerbated after exposure to light. Interestingly, a previous study has reported that priming increases resistance to biotic and abiotic stresses (Jisha *et al*., 2013). Based on our findings, we aimed to refine this statement. We hypothesize that priming diminishes the sensitivity to various environmental stresses by (translationally) upregulating genes that promote germination or downregulate genes that inhibit germination during the priming process. Such up‐/downregulation allows the seeds to pass through the phase in which they decide on whether to germinate or not during the priming process and avoids the inhibition of germination when these primed seeds are re‐imbibed in stressful environments. In natural conditions, seeds typically refrain from germinating in suboptimal environments. In theory, this enhanced resilience during germination might not necessarily improve the seedling's actual stress resistance. The diminished responsiveness to environmental stresses could, nonetheless, contribute to a reduction in longevity by promoting oxidative damage to molecular components. This could potentially elucidate the observed decrease in longevity during priming. On the contrary, the reduced longevity might also stem from the transcription and translation of new transcripts during the early stages of germination, particularly in hydropriming. These transcripts or proteins may not be present in dry mature seeds. Consequently, these newly introduced components could be more susceptible to damage during subsequent processes like drying or storage, potentially contributing to the observed decrease in seed longevity.

This study identified two phases of translational regulation changes during seed priming: the transition from the dry to stratified phases, characterized by genes predominantly showing translational upregulation, and the transition from the post‐light to primed phases, marked by genes mainly exhibiting translational downregulation. A novel finding is that many of the genes that were identified as translationally regulated during the shift from the dry to stratified stages are expressed as free transcripts in the dry seed (53%). This might concern a shift from free transcripts to monosomes. Earlier, it was shown that *c*. 50% of the mRNAs in dry seeds are bound to ribosomes; of these mRNAs, the majority is associated with monosomes (Bai *et al*., 2020). The same study found that 17% of these monosome‐associated mRNAs are translationally upregulated during seed germination, suggesting that part of the monosome bound mRNAs are primed to be translated during early germination. However, the study of Bai *et al*. (2017) excludes the translational regulation of non‐ribosome‐associated (free) mRNAs to monosomes since the polysome occupancy was calculated based on the total and polysome bound mRNA. Our data now also suggest that stored mRNAs that are not yet bound to ribosomes could be translated during early germination. It would be interesting to investigate whether these transcripts are more vulnerable to damage. Generally, mRNA lifespans are short as they are easily damaged and quickly turned over in cells. However, in seeds, mRNAs can survive for decades. Previous studies have hypothesized that it is the association with monosomes that ensures the survival of mRNAs during the dry stage (Bai *et al*., 2020). If indeed monosomes play a role in protecting mRNAs, then the high amount of polysomes in primed seeds might also play a role in the reduced longevity of primed seeds. One possibility is that the increased translation activity associated with polysomes leads to higher metabolic activity, which in turn could increase the rate of cellular processes including respiration and reactive oxygen species (ROS) production. ROS are known to cause oxidative damage to biomolecules, including mRNAs, which could lead to reduced mRNA stability and ultimately contribute to reduced seed longevity (Tanaka *et al*., 2007). Another possibility is the linkage of mRNAs with processing bodies (P‐bodies) and stress granules (SGs). While polysomes are typically associated with actively translating mRNA molecules in the cytoplasm, monosomes could potentially be involved in mRNA surveillance and protection mechanisms in various cellular compartments. mRNAs can shuttle between different cellular compartments, and their localization may be influenced by factors such as cellular stress. P‐bodies and SGs are cellular structures involved in mRNA metabolism and regulation, particularly under conditions of cellular stress. P‐bodies are sites of mRNA degradation and storage, while SGs are sites where translation initiation is inhibited, and mRNA molecules are sequestered under stress conditions (Decker & Parker, 2012). The presence of monosomes and polysomes could potentially influence the dynamics of P‐bodies and SGs, as these structures are intimately linked to mRNA stability.

Through the analysis of elevated mRNA–ribosome complex levels during hydropriming, we identified 30 genes. Subsequent protein interaction data analysis revealed RGGB's associations with key ribosomal and RNA‐binding proteins, suggesting a role for this protein in translation. RGGB functions as an mRNA binding protein (Bleckmann *et al*., 2023). RGGB is currently the sole gene in the list for which protein interactions have been identified. Notably, RGGB is upregulated at both the total RNA and ribosome‐associated RNA levels in primed seeds (Dataset S1). To investigate the effect of *RGGB* on seed germination, the *rggb* knock‐out mutant was analysed. The mutant exhibited slower germination than Col‐0, in both unprimed and primed seeds. This not only indicates a role of this gene in regulating the rate of germination but also shows that *RGGB* is not the key factor that determines the faster germination of primed seeds. An aberrant, but not further specified, germination phenotype was also reported for another T‐DNA line containing an insertion in RGGB (Warmerdam *et al*., 2019). Investigating the after‐ripening requirement of the *rggb* mutant revealed significantly higher dormancy levels (higher DSDS50) than Col‐0, highlighting its role in seed dormancy. Interestingly, this contrasts with findings of Sajeev *et al*. (2022) for *rggc*, which exhibited reduced dormancy (lower DSDS50), suggesting opposing functions of *RGGB* and *RGGC* in dormancy regulation. While priming improved germination performance across all lines, it also reduced seed longevity (p50) after controlled deterioration. *RGGB* does not seem to play a role in this since there were no significant differences between the longevity of the *rggb* mutant and the control lines. To further elucidate *RGGB*'s function, future studies could investigate its protein abundance and tissue‐specific localization. Additionally, identifying target mRNAs bound by *RGGB* and its homolog *RGGC* by, for example, RNA immunoprecipitation followed by sequencing or crosslinking immunoprecipitation could provide insights into *RGGB's* molecular roles in seed dormancy and germination regulation. Although further analyses are required, there is a strong indication that both *RGGB* and *RGGC* play a role in determining germination characteristics.

## Competing interests

None declared.

## Author contributions

PG and LB designed the experiments. PG performed the experiments with the help of BB, LLB, LAJW and AM and conducted the analyses. TWV and HN performed the data analysis of RNC‐seq and wrote the report on it. PG and LB wrote the manuscript.

## Disclaimer

The New Phytologist Foundation remains neutral with regard to jurisdictional claims in maps and in any institutional affiliations.

## Supporting information


**Dataset S1** Gene lists of differentially translationally regulated transcripts during seed priming.

**Fig. S1** Relative expression levels of *RGGB* in the T‐DNA mutant and transgenic line obtained by qRT‐PCR.
**Fig. S2** Phenotypic characteristics of primed seeds.
**Fig. S3** Polysome profiles and mRNA‐ribosome association during different stages of hydropriming.
**Fig. S4** Phenotypic characteristics of primed and unprimed Col‐0, the *rggb* mutant and the line containing the transgene *pRGGB:RGGB:GFP*.
**Notes S1** R scripts for DSDS50 and p50 calculation and R script for area calculation of polysome profiles.
**Table S1** Detailed list of all primers used in the cloning process.
Please note: Wiley is not responsible for the content or functionality of any Supporting Information supplied by the authors. Any queries (other than missing material) should be directed to the *New Phytologist* Central Office.

## Data Availability

The data that support the findings will be available in the Sequence Read Archive (SRA) under BioProject accession no. PRJNA1153751 https://submit.ncbi.nlm.nih.gov/subs/sra/ following an embargo from the date of publication to allow for the commercialization of research findings.
