# Supplementary material for: Unravelling the dynamics of seed‐stored mRNAs during seed priming
Source: New Phytol. 2025 Mar 28;247(5):2196–209. doi: 10.1111/nph.70098 (PMC12329163; doi:10.1111/nph.70098)
Supplement: Supplementary file 2 — Fig. S1 Relative expression levels of RGGB in the T‐DNA mutant and transgenic line obtained by qRT‐PCR. Fig. S2 Phenotypic characteristics of primed seeds. Fig. S3 Polysome profiles and mRNA‐ribosome association during different stages of hydropriming. Fig. S4 Phenotypic characteristics of primed and unprimed Col‐0, the rggb mutant and the line containing the transgene pRGGB:RGGB:GFP. Notes S1 R scripts for DSDS50 and p50 calculation and R script for area calculation of polysome profiles. Table S1 Detailed list of all primers used in the cloning process. Please note: Wiley is not responsible for the content or functionality of any Supporting Information supplied by the authors. Any queries (other than missing material) should be directed to the New Phytologist Central Office. [file NPH-247-2196-s001.pdf]

New Phytologist Supporting Information

Article title: Unravelling the dynamics of seed stored mRNAs during seed priming

Authors: Patricija Gran[1], Tessa W Visscher[2], Bing Bai[3], Harm Nijveen[4], Amir Mahboubi[5], Lars L Bakermans [6], Leo AJ Willems [7], Leónie Bentsink[8*]

Article acceptance date: 19 February 2025

Supplemental table 1

Supplemental figures 1-4

Notes S1

Supplemental table 1: Detailed list of all primers used in the cloning process

This table provides essential details for primers used in the cloning process. Target gene name (column 1), Target locus (column 2), Oligo sequence: Nucleotide sequence (column 3), Primer Length: Length of each primer in nucleotide units (column 4), Purpose and Adapter information (column 5 and 6) and description of the gene and primer combination (column 7).

Target gene name	Target locus ID	Oligo sequence (5'>3')	Length	Purpose	Cloning adapter	Description
**RGGB**	AT4G17520	GGGGACAAGTTTGTACAAAAAAGCAGGCTtacattagcatcaacggg	47	Gene amplification/ Cloning	Gateway AttB1	attb1 primer at4g17520 fw primer for cloning
**RGGB**	AT4G17520	GGGGACCACTTTGTACAAGAAAGCTGGGTcaaaatccaaaccgagtga	48	Gene amplification/ Cloning	Gateway AttB2	attb2 primer at4g17520 with stop codon for complementati on
**RGGB**	AT4G17520	GGGGACCACTTTGTACAAGAAAGCTGGGTActtacccaaagtagggaact	50	Gene amplification/ Cloning	Gateway AttB2	attb2 primer at4g17520 without stop codon for fusion expression
**RGGB**	AT4G17520	GGGGACCACTTTGTACAAGAAAGCTGGG	49	Gene amplification/ Cloning	Gateway AttB2	attb2 primer at4g17520 promoter:GFP fusion

	Ttgaatcgaaa<br>gagaaactgg

Supplemental figures

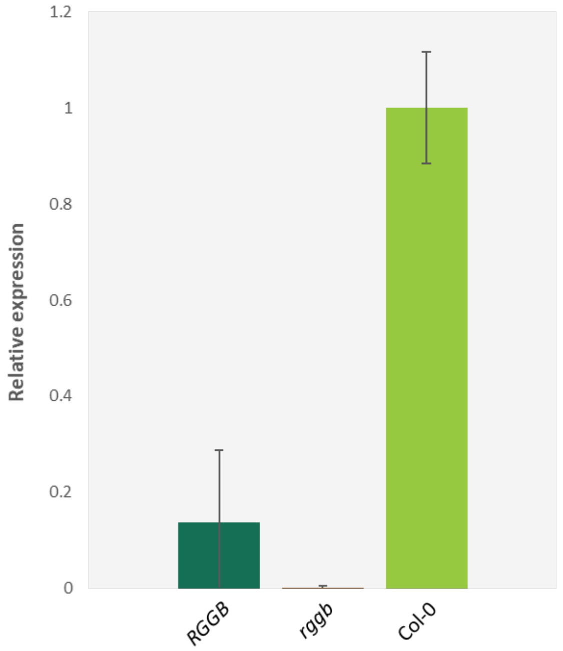

Supplemental figure 1: Relative expression levels of *RGGB* in the T-DNA mutant and transgenic line obtained by qRT-PCR. Relative expression levels of *RGGB* in primed Columbia (Col-0), the *rggb* mutant and the line containing the transgene *pRGGB:RGGB:GFP* (*RGGB*). The y-axis represents the relative expression level.

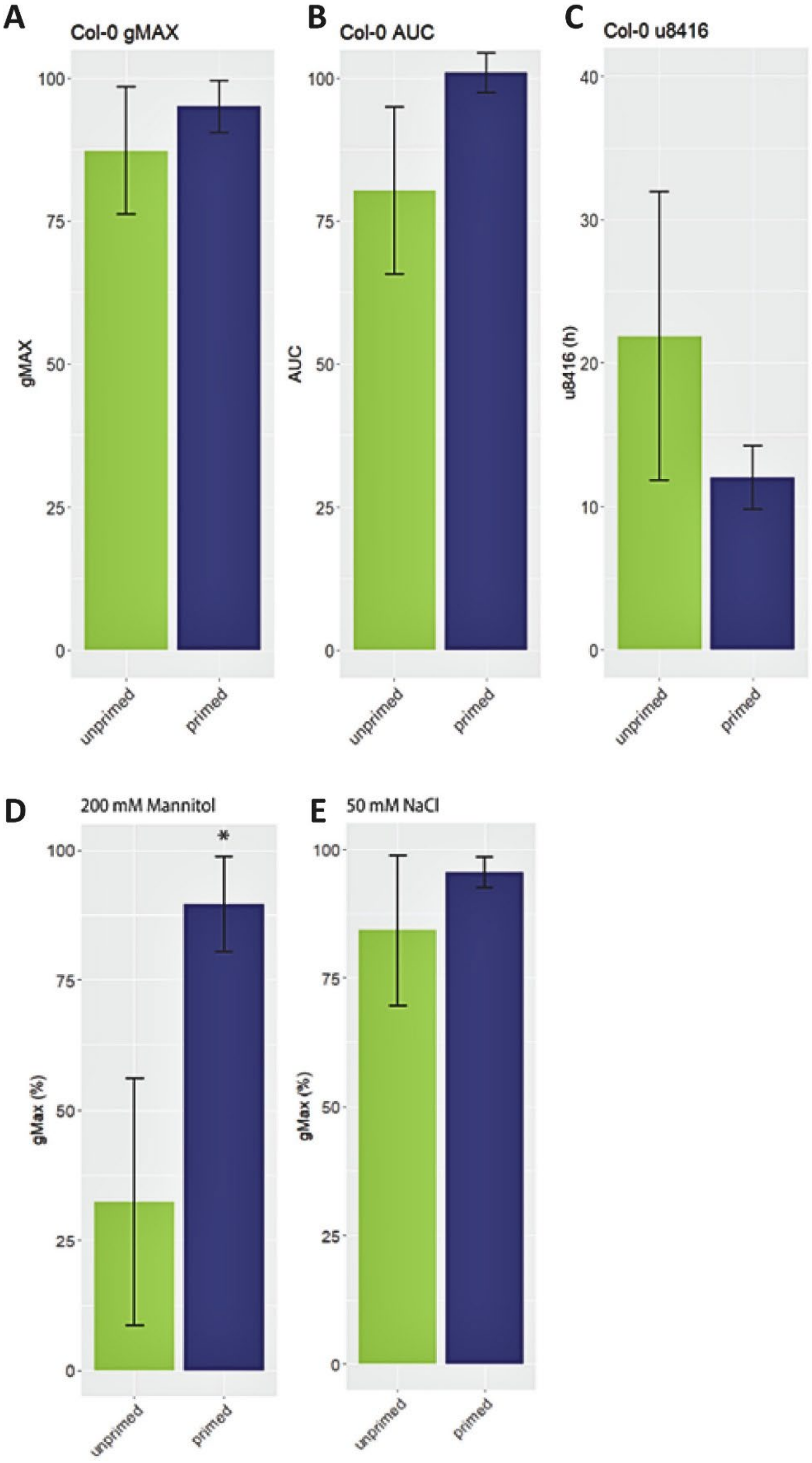

Supplemental figure 2: Phenotypic characteristics of primed seeds. A) Maximum germination (gMax), B) Area under the curve (AUC), C) Uniformity (time required to get from 16 to 84% germination; u8416), D) Maximum germination (gMax) in osmotic stress E) Maximum germination (gMax) in salt stress of unprimed and

primed Col-0 seeds. Mean values and standard errors (SE) are depicted based on four biological replicates. Asterisks denote significance levels determined through t-test. * indicates significance at $P \leq 0.05$.

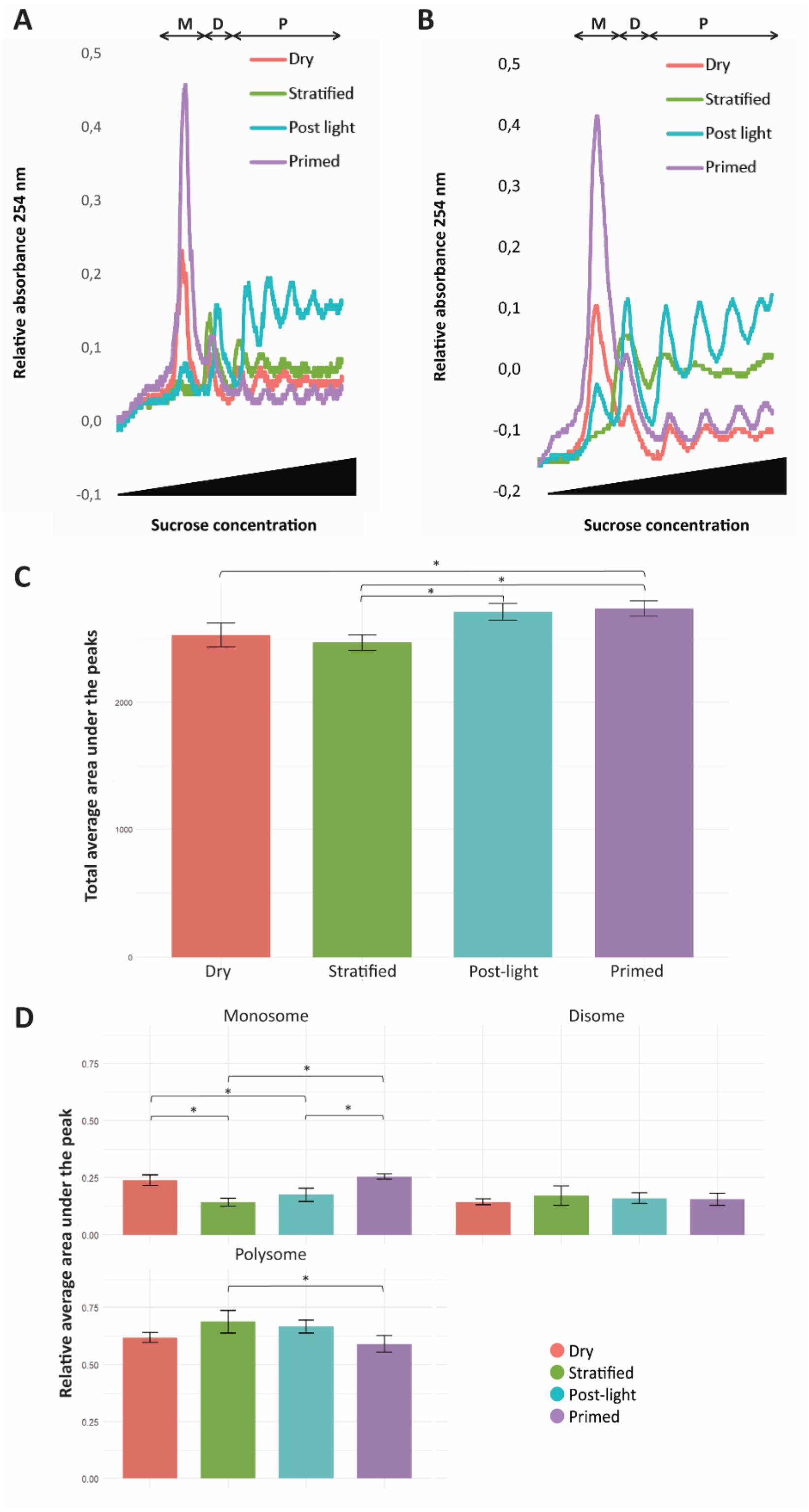

Supplemental figure 3: Polysome profiles and mRNA-ribosome association during different stages of hydropriming. Representative absorbance profiles of sucrose density gradient fractionated ribosomes for dry (salmon), stratified (green), post-light (turquoise) and primed (purple) seed samples. Peaks in the monosome area (M), disome area (D) and the polysome area (P) are presented. A) replicate 2, B) replicate 3. C) Total area under the peaks (monosome, disome, and polysome) across different priming stages, based on three biological

replicates. Statistical differences were determined using a two-way ANOVA. * Indicates a significant difference ($p < 0.05$). D) Relative average area under the monosome, disome and polysome peak, normalized across three biological replicates. Statistical significance was determined using a two-way ANOVA. * Indicates a significant difference ($p < 0.05$).

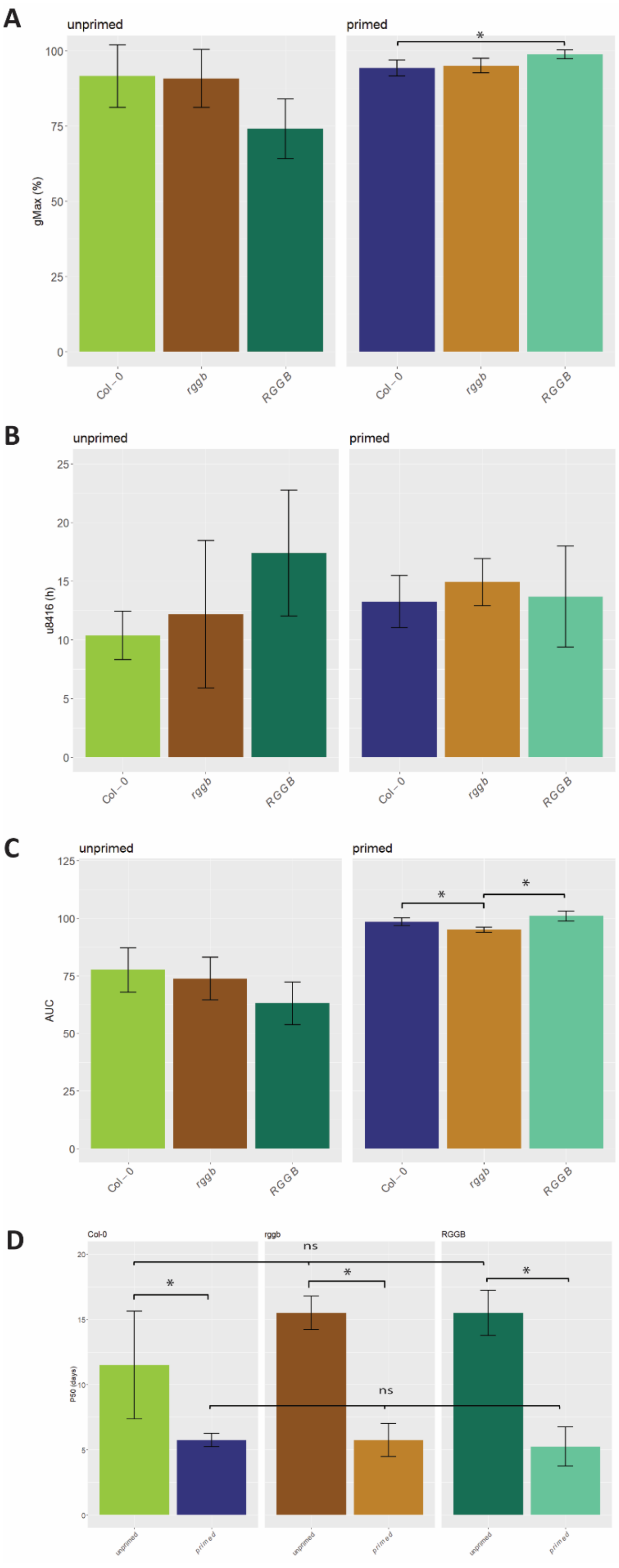

Supplemental figure 4: Phenotypic characteristics of primed and unprimed Col-0, the *rggb* mutant and the line containing the transgene *pRGGB:RGGB:GFP* (RGGB) . A) Maximum germination (gMax), B) Uniformity (u8416), C) Area under the curve (AUC), D) p50 of unprimed and primed Col-0, *rggb* and RGGB after Controlled

Deterioration Test (CDT) at 38°C and 75% RH (Righetti et al., 2015). p50 indicates the time (in days) when 50% seed viability is lost, calculated via a viability regression curve. Mean values and standard errors (SE) are depicted based on four biological replicates. Asterisks denote significance levels determined through t-test. * indicates significance at $P \leq 0.05$.

Notes S1

R scripts for DSDS50 and p50 calculation and for area calculation of polysome profiles

R script for DSDS50 and p50 calculation

```
#source("CurveFittingFunction-Arabidopsis.R")
source("CurveFittingFunction-Ara.R")

data.transformation.germination <- function(dm){
genotypes <- sort(unique(dm[,1]))
no.acc <- length(genotypes)
max.da.acc <- max(table(dm[,1]))

looking.for.na <- which(dm[,3]==999)
if(length(looking.for.na)>0) dm[looking.for.na,3] <- NA

da <- array(dim=c(no.acc, max.da.acc, 3), NA)

for(i in 1:no.acc){
da[i,,1] <- genotypes[i]
da[i,1:table(dm[,1])[i],2] <- dm[which(dm[,1]==genotypes[i]),2]
da[i,1:table(dm[,1])[i],3] <- dm[which(dm[,1]==genotypes[i]),3]
}
return(da)
}

mfg.curve <- function(da){

xmin <- min(da[,,2], na.rm=TRUE)
xmax <- max(da[,,2], na.rm=TRUE)

model.curve <- curve.fitting.ara(da, x.min=xmin, x.max=xmax)
fitted.v <- results.curve.fitting.ara(da,model.curve, x.min=xmin, x.max=xmax)$fitted.val
grid.c <- results.curve.fitting.ara(da,model.curve, x.min=xmin, x.max=xmax)$gri

return(list(modell=model.curve, fiv = fitted.v, gri=grid.c))
}

results.germination <- function(da, mofigri){

#If you want to calculate the DSDS75 or so, you can play here with the numbers
wi50 <- function(x) which.min(abs(x-50))
hm50 <- function(x) abs(x-50)[which.min(abs(x-50))]

ii <- apply(mofigri$fi, 1, wi50)
alk <-apply(mofigri$fi, 1, hm50)

DSDS50.day <- NULL
for(i in 1:dim(da)[1]){
DSDS50.day[i] <- mofigri$gri[ii[i]]
}

genotypes <- unique(da[,1,1])

special.acc.test <- genotypes[which(alk>2.5)]
#where the closest to 50% is still more than 2.5% away from 50% -> indicating that
#probably it did not reach the maximum

mxma.test <- apply(mofigri$fi, 1, max)
wi47.5 <- genotypes[which(mxma.test < 47.5)] #accessions that did not reach 50% level - 5%
```

```
return(list(D50=cbind(genotypes,DSDS50.day), special=special.acc.test, under475=wi47.5))

}

model.results <- function(da){
momo <- mfg.curve(da)
resi <- results.germination(da, momo)
resi$fi <- momo$fi
resi$gri <- momo$gri

return(resi)
}


plot.overview.pdf <- function(da, resi, filename){
pdf(filename, paper="a4r", width=11, height=8)
par(mfrow=c(5,5), mar=c(2,2,2,2))

genotypes <- unique(da[,1,1])
xmin <- min(da[,,2], na.rm=TRUE)
xmax <- max(da[,,2], na.rm=TRUE)

no.pa <- ceiling(length(genotypes)/25)

plot.coll <- matrix(c(1:length(genotypes), rep(NA, no.pa*25-length(genotypes))), no.pa, 25, byrow=TRUE)

plot.col <- matrix(rainbow(25), no.pa, 25, byrow=TRUE)

# if you want to calculate the DSDS75 for example, you can change here where the program draws the line
for(v in 1:no.pa){
for(k in 1:25){
  x <- da[plot.coll[v,k],,2] #NL1999data[plot.coll[v,k],,1]
  y <- da[plot.coll[v,k],,3]
  plot(x,y, xlim=c(xmin,xmax), ylim=c(0,100))
  lines(resi$gri, resi$fi[plot.coll[v,k],], col=plot.col[v,k])
  abline(h=50, col="gray")
  abline(v=resi$D50[plot.coll[v,k],2], col="gray")
  text(x=xmin, y=95, label=paste("acc.no",genotypes[plot.coll[v,k]]), pos=4, cex=0.85)
}
}
dev.off()

}
```

R script for area calculation of polysome profiles

```
# set working directory
setwd("C:/Users/gran001/OneDrive - Wageningen University & Research/AUC profiles")

# Load necessary libraries
library(tidyverse)
library(readxl)
library(pracma)

# Read the specific sheet
profile1 <- read_excel("Polysome profiles Patricija.xlsx", sheet = "Profile 1")

# Determine the minimum value within the dataframe
min(profile1)

# Add a positive number to the whole dataframe
profile1 <- profile1 + 2

# Add a new column called "Serial" with row numbers
profile1 <- profile1 %>%
        mutate(Serial = row_number(),.before = 1)




# Define x-axis ranges for segmentation of the monosome, disome, polysome, and total areas, respectively
profile1Ranges <- list(c(278, 548), c(548, 751), c(751, 1559), c(278, 1559))


# Create a line plot
plot1 <- ggplot(profile1, aes(x = Serial, y = Dry)) +
      geom_line(color = "blue") +
      labs(title = "Segmented Line Plot", x = "Sequence", y = "Value") +
      theme_minimal()

# Calculate AUC for each range
auc_results <- lapply(profile1Ranges, function(range) {
  # Filter data for the current range
  segment <- profile1 %>% filter(Serial >= range[1] & Serial <= range[2])

  # Calculate AUC using trapezoidal rule
  auc <- trapz(segment$Serial, pmax(segment$Dry, 0))

  # Return results
  list(range = range, auc = auc)
})

# Print AUC results
for (i in seq_along(auc_results)) {
  cat(sprintf("Range %d-%d: AUC = %.4f\n",
          auc_results[[i]]$range[1],
          auc_results[[i]]$range[2],
          auc_results[[i]]$auc))
}

# Add vertical lines to show segmentation
for (range in profile1Ranges) {
  plot1 <- plot1 + geom_vline(xintercept = range, linetype = "dashed", color = "red")
}

# Display the plot
print(plot1)
```
